# Mechanisms by which phytogenic extracts enhance livestock reproductive health: current insights and future directions

**DOI:** 10.3389/fvets.2025.1568577

**Published:** 2025-04-16

**Authors:** Adedeji O. Adetunji, Jacqueline Price, Henrietta Owusu, Esiosa F. Adewale, Precious Adedayo Adesina, Tolulope Peter Saliu, Zhendong Zhu, Christian Xedzro, Emmanuel Asiamah, Shahidul Islam

**Affiliations:** 1 Department of Agriculture, University of Arkansas at Pine Bluff, Pine Bluff, AR, United States; 2 Department of Biology, University of Louisville, Louisville, KY, United States; 3 Graduate School of Integrated Sciences for Life, Hiroshima University, Higashi-Hiroshima, Japan; 4 Department of Physiology, College of Medicine, University of Kentucky, Lexington, KY, United States; 5 College of Animal Science and Technology, Qingdao Agricultural University, Qingdao, China; 6 Laboratory of Food Microbiology and Hygiene, Hiroshima University, Higashihiroshima, Japan

**Keywords:** plant extract, hormone regulation, immune modulation, oxidative stress mitigation, reproductive health, phytogenic extracts, bioactive compounds, livestock reproduction

## Abstract

Reproductive health is a critical determinant of livestock productivity and economic sustainability. However, it is often compromised by infectious diseases, environmental stressors, and nutritional deficits. Phytogenic extracts—bioactive compounds derived from medicinal plants—have emerged as sustainable alternatives to synthetic antibiotics and hormones, exhibiting antimicrobial, antioxidant, and immunomodulatory properties. These extracts influence key reproductive processes such as follicular development, oocyte maturation, and endometrial health while mitigating the detrimental effects of oxidative stress and pathogenic infections. Recent findings suggest that phytogenic extract can enhance reproductive performance, improve oocyte quality, and support pregnancy outcomes. Despite the growing body of evidence, optimal application strategies and the full breadth of their biological effects remain insufficiently explored. This review focuses on the molecular mechanisms modulated by phytogenic extracts, particularly in the context of hormone regulation, immune modulation, and oxidative stress mitigation. We also identify critical knowledge gaps and propose future research directions to optimize the use of phytogenic extracts as a sustainable approach to enhancing livestock reproductive health.

## Introduction

Phytogenic extracts are plant-derived compounds incorporated into livestock feed to improve feed quality and livestock production (1). These natural compounds have been explored as alternatives to antibiotic growth promoters in recent years due to their antimicrobial and antioxidative properties (2). In addition, public health concerns over antimicrobial drug resistance associated with using antibiotics in maintaining gut health, promoting growth, and limiting foodborne pathogens are alarming (3, 4). On this note, plant extracts and their bioactive compounds, such as carvacrol, eugenol, thymol, capsaicin, cineole, quercetin, and gingerol, which exhibit antibacterial, antifungal, antiviral, and anticoccidial properties, have been used as alternatives (5). Phytogenic feed additives (PFAs) indirectly improve the production and performance of commercial livestock by enhancing animal’s utilization of nutrients from feed. More so, they contain multi-component agents that help perform immunomodulatory activities and prevent the establishment of infections in both humans and livestock (6). As a result, PFAs have been leveraged in ameliorating and mitigating reproductive, digestive and nutritional disorders.

Among several factors, hormone regulation, immune modulation, and oxidative stress mitigation are key determinants of male and female reproductive success. Hormones such as Gonadotropin-releasing hormone, testosterone, estrogen, progesterone, luteinizing hormone (LH), and follicle-stimulating hormone (FSH) play a critical role in the endocrine regulation of reproductive processes, from attaining sexual maturity, estrus, steroidogenesis, ovulation, fertilization and corpus luteum formation (7, 8). These reproductive processes are regulated by the phosphatidylinositol 3-kinase (PI3K)/protein kinase B (AKT) mechanistic target of the rapamycin complex1 (mTOR) pathway. Specifically, FSH is involved in the activation of the PI3K pathway. It is involved in follicular growth from primordial follicles to antral follicles (9). The role of LH has been extended beyond triggering the release of mature follicles to determining the number of recruitable antral follicles (10). Reactive oxygen species (ROS) is a byproduct of physiological processes of oxygen consumption biological systems, and they mediate inter- and intra-cellular signaling. In addition, ROS is required for sperm capacitation and acrosome reaction (11). However, the accumulation of ROS due to the imbalance between antioxidants and oxidants causes oxidative stress which has a deleterious effect on sex cells (12, 13). Lastly, inflammation is required in reproductive processes such as ovulation, corpus luteum formation, and luteolysis (14). However, recurrent or persistent inflammation from bacteria or viral challenges can cause deleterious effects, impairing oocyte development and quality as well as embryo development. Recognition of bacteria or viruses by toll-like cell receptors triggers several signaling cascades through the FκB or MAPkinase pathways, resulting in the recruitment of Tumor Necrosis Factor α–TNFα, interleukin–IL 1 and 8. The recruitment of these proinflammatory cytokines and chemokines results in cellular injuries and negatively impacts reproductive outcomes (15).

Furthermore, the role of PFAs in ameliorating and mitigating reproductive disorders has been widely reported. For instance, Singh et al. (16) reported that garlic, black cumin, and turmeric affect steroid hormone precursor cholesterol when supplemented in White Leghorn diet during the laying phase. Besides reducing total serum cholesterol and malondialdehyde, dietary phytogenic extracts also modulate the immune response by increasing immunoglobulin G (IgG) and immunoglobulin A (IgA) levels (17). Another study showed that polyherbal formulations composed of ginger rhizome, onion, and moringa could trigger estrus response and ovarian luteal activity in dairy cows (18). Plant extracts containing catechin, gallic acid, and procyanidins have been reported to scavenge accumulating ROS, hereby proffering oxidative stress mitigation effect (19). In this review, we summarized the role of PFAs in mitigating livestock reproductive diseases and discussed their potential mechanisms of action. While other studies have focused on promoting livestock growth performance and health with the use of plant extracts, we aimed to provide a comprehensive overview of current research on the role of PFAs in improving reproductive outcomes in the context of hormone regulation, immune modulation, and oxidative stress mitigation.

## Infertility and pelvic inflammatory diseases in livestock

**Figure 1:**
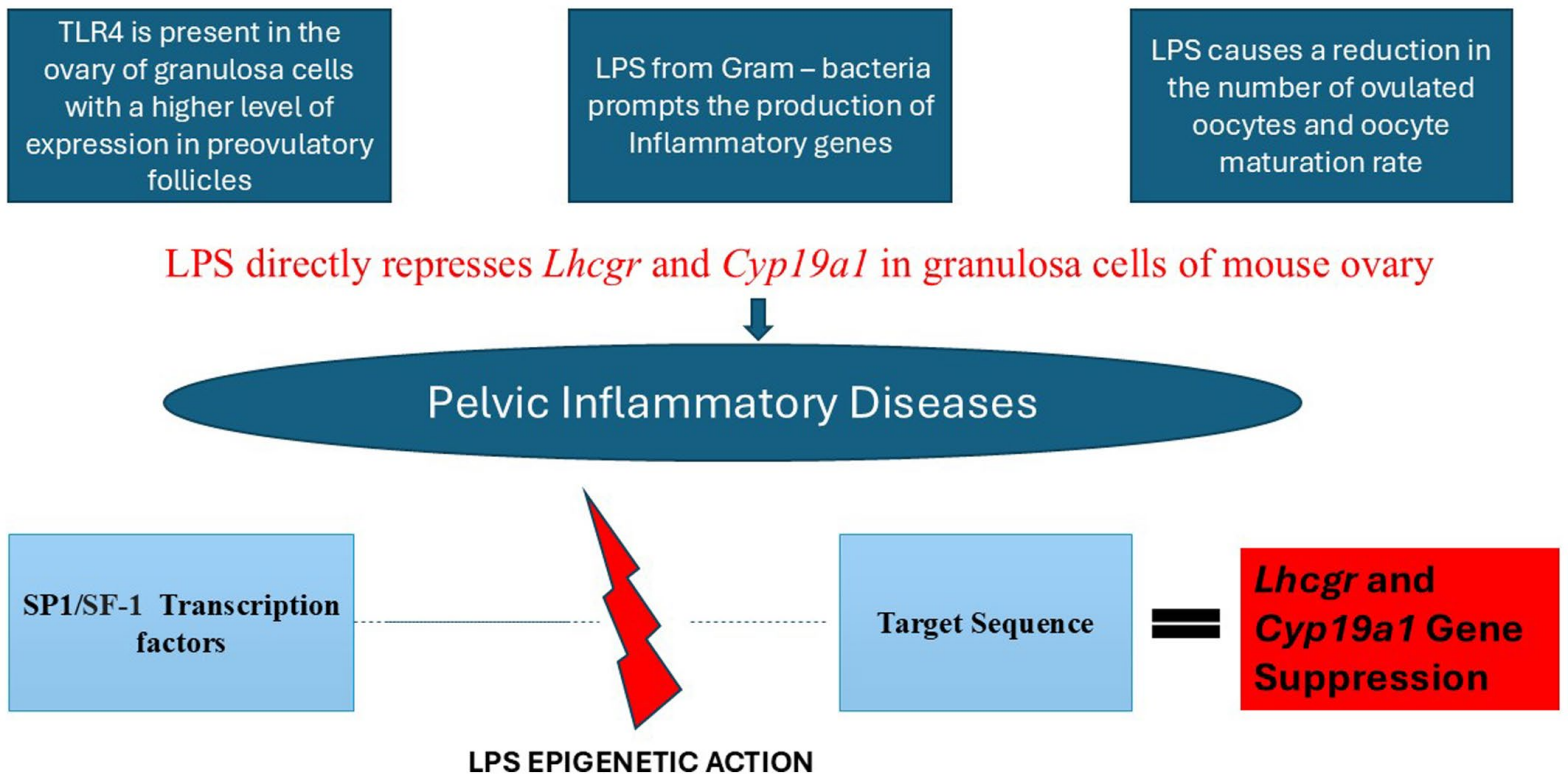
Mechanism by which bacteria influence reproductive outcomes.

A livestock population’s reproductive success largely determines the efficiency of animal production; therefore, disease prevention and control are integral to reducing the risk of infertility in animals. However, infertility and pregnancy attrition are associated with pelvic inflammatory diseases in livestock. Prominent anaerobic bacteria, including *Escherichia coli*, *Trueperella pyogenes*, *Prevotella melaninogenica*, *Staphylococcus* spp., and *Fusobacterium necrophorum* cause uterine disease (such as metritis, endometritis, and mastitis) in ruminant livestock, specifically in ruminants such as dairy cows (20). More so, *Campylobacter* and *Aliarcobacter* are zoonotic diseases that have been found to colonize the uterus, intestine, and gall bladder, and they have been associated with abortion and infertility in ruminants (21). Similarly, infections by *Chalymidia*, *Ureaplasma* and *Mycoplama* spp., such as *M. bovis*, *M. genitalium*, and *M. hominis* cause morphological and physiological changes to the sperm as well as cumulus-oocyte complex incompetence in both livestock and human (21–23). Pattern recognition molecules are found on cell surfaces or specific organelles along different molecular pathways, such as the bovine uterus; therefore, their effect range occurs in different body areas. Inflammatory mediators produced from the pattern recognition receptors and bacterial products can transfer to the animal’s ovaries via general circulation or countercurrent vascular mechanisms that carry endometrial prostaglandins to the ovary (7). Therefore, inflammation and the endotoxins of bacterial products in the ovary damage ovarian follicular function; the meiotic development of oocytes, the ability of an oocyte to fertilize, and mitotic development after fertilization are diminished (24). The innate immune response associated with inflammation inflicts endometrial cell damage (as well as the endotoxins released from cells), directly linking inflammation with an increased risk of infertility. Another study suggests that ovarian follicular cysts, characterized by bacteria infection in humans and animals, are closely connected to the Lhcgr promoter region’s methylation level (7) (Figure 1).

Apart from pathogenic organisms, welfare disorders such as housing conditions, environmental pollution, and hygiene cause metabolic, heat, oxidative stress, and lameness in cattle (25). The overall effect of these conditions includes a reduction in the production of oocytes (25), loss of embryo (26), asymptomatic estrus (27), and reduction in ovarian size (28). In addition, exposure to these welfare disorders causes inflammation accompanied by oxidative stress. Reactive oxygen species, superoxide anion, hydroxyl radical, peroxyl radical, alkoxyl, and hydroperoxyl are highly reactive/unstable because of their unpaired electrons and are stabilized with the acceptance of electrons from other molecules (24). In addition, bacterial infection has been reported to be metabolically costly, causing an increase in oxidative stress, and it also results in the diversion of energy from normal developmental processes to potentiate disease resistance mechanisms (29). Although important in normal reproductive physiology, in the event of pathogenic infections in the reproductive system, ROS production can be heightened to a level of oxidative stress. This may damage oocytes after prolonged exposure (30). The cumulative effects of pathogenic toxins, inflammation, and oxidative stress result in deteriorating oocyte quality, which is usually associated with reproductive aging in livestock (24). Research further suggests that inflammation and oxidative stress reduce the pool of primordial follicles by prematurely inactivating them, shortening the reproductive life span (31). This phenomenon has been observed in dairy cows, rodent models, and humans (24).

## Phytogenic extracts and bioactive compounds

Throughout history, plants and their extracts have been utilized for their therapeutic qualities. Although the focus of this application has frequently been on human health, plant extracts have been employed in animal health management and ethnoveterinary therapy (32). According to the world’s flora status, there are around 391,000 plant species. Between 35,000 and 70,000 plant species are thought to have been utilized medicinally (33). Research on plant bioactive compounds is underway, especially in plants containing tannin and saponin metabolites (34). Methane generation, foam formation/bloat management, milk and meat quality enhancement, and reproductive efficiency are some of their applications (35). To provide primary medical care for animals, the World Health Organization (WHO) has acknowledged the need to investigate and mobilize ancient medicinal practices. The WHO also recognizes that traditional medicine may be crucial to the development of livestock in third-world countries (36). These chemical-containing phytogenic plants are categorized as feed additives because they cause physiological reactions in the animal body (37). According to Sarswat and Purohit (38), medicinal herbs can effectively treat reproductive problems, enhancing livestock’s reproduction capacity. Bioactive compounds in plants can affect nearly every stage of the reproductive cycle, from gamete generation to puberty regulation. They can have both positive and negative effects on male and female reproduction. These bioactive compounds can also enhance sensitivity to sex hormones (39) and promote the expression of male reproductive behaviors, such as wooing and mating behavior (40). Traditional veterinary medicine has documented using plants to induce estrus in ruminants and as general fertility enhancers (18). Plant species that possess chemicals with steroid-like action are an essential group. Certain plants are known to induce chickens to lay more eggs, indicating that they influence poultry ovulation (41). Traditional veterinary medicine has utilized several plants to treat parturition-related issues, including dystocia, placenta expulsion, and recuperation from challenging parturition (42).

In traditional animal and alternative medicine, plants and their extracts are also well-known phytobiotics or phytogenics (43). According to Fasinu et al. (44), the mechanisms of action of herbs include changes in renal excretion of herbs and their metabolites, induction and inhibition of metabolic enzymes and transport proteins, and modification of gastrointestinal functions with resulting effects on herb absorption. Plant extracts can be produced or extracted from plants using solvent extraction, steam refining, maceration, and cold squeezing (45). However, continued research has shown that secondary metabolites’ origin, chemical characteristics, dietary concentration, and feed quality determine whether they have a positive or negative effect. Accordingly, findings from various *in vivo* and *in vitro* tests involving dairy cattle, sheep, and goat point to the possibility of incorporating limited amounts of secondary metabolites into the diet to enhance feed utilization. A potential strategy to lessen the detrimental effects of heat stress in livestock is dietary intervention, such as adding PFAs to diets (46). Because they are inexpensive, readily available, safe, and may have antioxidant qualities against heat stress, PFAs have received much attention. Compared to veterinary medications, plant-based extracts are more readily available and safer to use (47). According to recent research, many antimicrobials and antiparasitic medications are derived from bioactive chemicals found in plants (48). Certain bioactive substances, including polyphenols, flavonoids, terpenes, tannins, vitamin C, essential oils, and carotenoids, may be responsible for their antibacterial properties. The anti-inflammatory and other therapeutic properties of plant-based bioactive compounds have been extensively researched in recent years, both *in vitro* and *in vivo*. Understanding the anti-inflammatory mechanisms that trigger the innate immune response to infections or injury requires the use of plant-based bioactive chemicals to modify the inflammatory response (49).

## Applications of phytogenic extracts in livestock health and performance

Animal feeds include large concentrations of plant extracts with potent anti-inflammatory, immunomodulatory, antioxidant, and metabolism-regulatory effects. Changes in the gut microbiota, higher nutrient digestibility and absorption, greater nitrogen absorption, enhanced immunological response, and antioxidant activity are potential methods by which the growth-promoting herb acts in the animal. In addition, extracts with phenolic compounds perform their oxidative function by donating hydrogen atoms to metals, causing a reduction in pro-oxidant activities (50). Moreover, they improve antioxidant function in animal tissues by quenching oxygen and reducing peroxide generation. These phenolic compounds also modulate the gut microbiota and regulate Nrf2 antioxidant and inflammatory pathways (51). Because of the similar structure of isoflavones, polyphenols interact with estrogen receptors and regulate transcription factor activator protein (AP-1) and nuclear factor kappa B (NF-κB) (52). Besides, saponins modulate vascular remodeling and proliferation via NF-κB downregulation by inhibiting the expressions of VE-cadherin and VEGFR-2 complexes (53). According to Iranparast et al. (54), these compounds work by damaging the glycolipid walls of bacterial cells, which causes leakage and a decrease in cytoplasmic compositions. More so, ginger root extract containing shogaols and gingerols reduces oxidative stress and enhances mucosal development (55). In addition, the role of plant extracts such as ginger root, onion peel, and green tea in improving immune function, gastrointestinal health, and overall animal performance cannot be overemphasized. The use of selected phytogenic extracts has been reported to reduce the expression level of toxin-encoding genes associated with necrotic enteritis in broiler chickens (2). Similarly, ginger root supplementation up to 1.5% in broiler chicken lowered oxidative stress, promoted mucosa growth and boosted overall growth performance (56). Plant herbs have potent antimicrobial activity, particularly against Gram-negative and Gram-positive bacteria, such as *Salmonella*, *Escherichia*, *Staphylococcus*, *Klebsiella*, and *Proteus* (20). Pigs and poultry have been shown to benefit from phytogenic effects in feed palatability, gut function, endogenous enzyme secretion, nutrient digestibility, gut microbiota, immune function, and growth promotion (5). Certain bioactive substances, including polyphenols, flavonoids, terpenes, tannins, vitamin C, essential oils, and carotenoids, may be responsible for their antibacterial properties. The anti-inflammatory and other therapeutic properties of plant-based bioactive compounds have been extensively researched in recent years, both *in vitro* and *in vivo* (49). According to Liu et al. (57), giving plant extracts to weaned pigs decreased inflammation and diarrhea caused by an *E. coli* infection and increased expression of genes linked to immune responses. In a study based on the use of ginger extracts in drinking water, Arshad et al. (58) observed similar results, showing better immunity and growth performance of commercial broiler chicks. Lee et al. (59) found that adding ginger extracts to sow mixtures improved the amount of immunoglobulin in the sow colostrum, which enhanced the piglets’ immunological function. *Echinacea purpurea*, or purple coneflower, is a popular and significant herb with an immunostimulant effect. According to research by Dehkordi et al. (60), feeding chicken broilers *Echinacea purpurea*, especially for an extended period, may boost immunity and performance. Böhmer et al. (61) also observed that laying hens and fattening piglets exhibit immune-stimulating effects when *Echinacea* juice is applied repeatedly for brief periods. Similarly, the legume family, alfalfa (*Medicago sativa*), has a multifaceted impact on the body, supporting detoxification, cleansing, and nutrient intake and absorption while boosting the immune system due to its diverse chemical makeup. Alpha-carotene, beta-carotene, beta-sitosterol, chlorophyll, coumarin, cryptoxanthin, daidzein, fumaric acid, genistein, limonene, lutein, saponins, stigmasterol, and zeaxanthin are among the valuable phytochemical substances found in it (62). Alfalfa extract enhanced the immunological response in broiler chicken mixtures without negatively impacting performance (63). Pietrzak and Grela’s research also supported the beneficial impact of alfalfa extract on the rise in fatteners’ lymphocyte and total white blood cell counts (64). According to Zhang et al. (65), one way in which saponins work is by making the intestinal mucosa more permeable, which increases the absorption of viral antigens.

Furthermore, the effect of plant extracts on reproductive outcomes has been the focus of numerous studies. Ros-Santaella and Pintus demonstrated that the aqueous extract of *Rhodiola sacra* improved the biochemical and sperm properties of cryopreserved swine sperm (66). Furthermore, adding some phytogenics to ruminant diets may improve reproductive efficiency since reproductive performance is the main indicator of livestock production systems’ success (67). Research has demonstrated that both fresh and post-thawed semen benefit from extracts of herbs or phytochemical substances (68). While some plant extracts, including extracts of *Tribulus terrestris* in rams (69), enhance sperm counts or alter the components of sperm plasma, like *Acacia*, others reduce sperm production, primarily by harming germ cells. Antioxidant chemicals in bitter kola have been linked to tissue improvement and increased testosterone levels (70). Bioactive compounds such as diterpenes, triterpenes, flavonoids, polyphenols, and sesquiterpenes provide rosemary (*Rosmarinus officinalis*) with antioxidant qualities. According to reports, the plant possesses aphrodisiac properties in humans and animals (71). Furthermore, according to De Jong et al. (72) and Kim et al. (73), several plants with antioxidant qualities have been reported to improve nitric oxide generation and immunological function. Ginseng has been used since ancient times in China to improve their animals’ sexual behavior and heal sexual problems. To improve the outcome of *in vitro* fertilization procedures, PFAs containing antioxidants have been widely used in several species to lower the production of oxidative stress in the medium (73). The primary defense mechanisms of biological molecules against oxidation are photogenic antioxidant qualities, which are based on their capacity to supply electrons or hydrogen ions and delocalize unpaired electrons within the phenolic aroma ring of their structure (74). Rams given *Moringa oleifera* leaf extract orally (40 mg/kg body weight) showed improvements in semen volume, sperm concentration, sperm motility, viability index, membrane integrity, ascorbic acid, total antioxidant capacity, seminal plasma catalase, superoxide dismutase, glutathione reductase, and peroxidase activities, as well as some metabolite enzymes, such as alkaline phosphatase and acid phosphatase (75). According to Pamungkas et al. (76), bulls fed herbs for 3 months showed increased sperm volume, concentration, viability, and motility. Furthermore, it was observed that boars given ginseng root had superior levels of antioxidant enzymes, including glutathione peroxidase and antioxidant levels in their seminal plasma, as well as much higher sperm concentration and significantly lower lipid peroxidation (77). According to a recent study by El-Azrak et al. (78), rams given cinnamon oil orally showed enhanced semen quality and heightened libido. Furthermore, when buffalo spermatozoa were supplemented with 1% doses of green tea extract, which is high in catechins, the *in vivo* fertility rate increased by 34.21% compared to the control treatment (79). Compared to the control, pregnant ewes given baseline diets supplemented with 5 g of propolis exhibited a substantial rise in leucocytes and a decrease in erythrocytes, as well as mean corpuscular hemoglobin, according to Shedeed et al. (80). Supplementing the nursing dairy cow’s basic diet with 60 g of *Moringa oleifera* leaf meal per cow per day resulted in a significant increase in serum total antioxidant capacity, total protein, and IgG while lowering levels of non-esterified fatty acids (81). Additionally, adding yucca to the diets of dairy goats (82) and cattle (83) improved the conception rate, shortened the estrus cycle, and raised goats’ fertility and kidding rates. Ginger and *Echinacea* extract improved the acrosome integrity, mitochondria function and lipid peroxidation owing to their antioxidant properties (84). By administering 25 mL of sterile hydromethanolic extract of *Azadirachta indica* or 20 mL (10 mg/mL) of sterile hydromethanolic leaf extract of *Achyranthes aspera* (200 mg) intrauterine for 3 days in a row, Nikhade et al. (85) evaluated the effectiveness of herbal extracts in treating subclinical endometritis disorder in cows. After no therapy, their results showed that *A. aspera* had a higher curative efficacy than *A. indica*. Compared to 20% for the control treatment, the conception rates for *A. indica* and *A. aspera* were 50 and 40%, respectively. Furthermore, the effect of *Moringa oleifera* extract in raising calcium ions and modifying the expression of genes linked to fertility in sheep oocytes may also be responsible for this improvement (86). Additionally, Barakat et al. (87) found that adding 0.3 mg/mL of green tea extract to the *in vitro* maturation medium greatly improved sheep oocyte maturation and embryo development. The performance and immunological function of laying hens were significantly enhanced by dietary combinations of plant extracts and probiotics (88). Numerous factors, such as the type and section of plant utilized, harvest season, procedures for preparing phytogenic additives, and herbal extraction techniques, may be responsible for the variations in the results (89) (Table 1).

**Table 1:** Representative phytogenic extracts that influence reproductive health of livestock.

Health/reproductive condition	Herb used for mitigation	Dosage	Route of administration	Species	Action	References
Delayed puberty	*Aegle marmelos* and *Murraya koenigii* supplementation	5 g per day for 9 days	Oral supplementation	Buffalo heifers	Improved estrus induction and ovulation rates	(112)
Poor semen quality	Turmeric, garlic, and moringa extracts	3 g per kg of diet	Oral supplementation	Rabbit bucks	Increased sperm motility and viability	(115, 116)
Infertility	*Momordica charantia L*. intrauterine administration	1.5 mL orally for 8 weeks	Oral supplementation	Cows	Improved pregnancy rates and fertility outcomes	(117)
Low estrus and conception rates	*A. marmelos* and *M. koenigii* supplementation	5 g per day for 9 days	Dietary supplementation	Buffalo heifers	Higher estrus response and better conception rates	(112)
Oxidative stress and inflammation	Various phytogenic extracts	50 mg/kg body weight	Feed additive	Rabbit bucks	Reduced oxidative stress and improved immune function	(115, 116)
Low ovulation rates	β-carotene supplementation	50 mg per day	Dietary supplementation	Goats	Increased progesterone synthesis and higher ovulation rates	(114)
Immune deficiency and low milk yield	Propolis supplementation	5 g per day	Dietary supplementation	Sheep	Improved milk composition and immune response	(80)
Low sperm quality and viability	Ginseng root supplementation	Root extract in diet for 8 weeks	Dietary supplementation	Boars	Enhanced sperm viability and testosterone levels	(77)
Low immunity and poor gut health	*Echinacea purpurea* feed additive	1% dietary supplementation	Dietary supplementation	Chickens	Boosted immune system and reduced disease susceptibility	(60)

## Mechanisms of phytogenic extracts in reproductive health enhancement

### Gut health enhancement and nutrient absorption

The gastrointestinal tract, being the largest and most exposed surface of the body, plays a crucial role in nutrient absorption and houses a complex chemosensory system with numerous nerves and receptors. Phytogenic extract enhances gut health and improves nutrient absorption and uptake, which is essential for reproductive health by promoting the population of beneficial bacteria while reducing pathogenic ones and modulating gut microbiota (90, 91). A study demonstrated that pigs fed a diet supplemented with 5 g/kg of *Allium* extract showed improved growth performance, as evidenced by increased average daily gain (ADG). The addition of *Allium* extracts also positively improved gut health and nutrient absorption and reduced harmful bacteria like *Salmonella* spp. and *Clostridium* spp., while boosting beneficial *Lactobacillus* spp. counts (92). Similarly, the use of allium extract as a supplement in laying hen feed improved gut microbiota, the number of beneficial bacteria, and overall growth rate (93). This phytogenic extract-induced modulation of the intestinal microbiota is essential because a balanced gut microbiome enhances nutrient absorption and reduces inflammation, both of which directly impact reproductive efficiency (94). Specifically, Laguardia-Nascimento et al. (95) highlighted the role of microbiome-gut-reproductive axis in maintaining the reproductive axis. This study shows that bacteria from the gut can be carried via hematogenous pathways to the reproductive tract. Thus, the gut microbial population influences the microbiome population in the cervix, vaginal, ovary, and uterus (95, 96). Likewise, it has been reported that bacteria population such as *bateriodes, actinobacteria* and *streptococcus* influences the level of circulating follicle-stimulating hormone (FSH) and luteinizing hormone (LH), which are hormones known to regulate ovulation and follicle development (97). Overall, these beneficial bacteria improve feed intake and overall digestive health. Furthermore, by reducing the population of harmful bacteria, phytogenics can decrease the production of toxic substances in the gut that may negatively impact reproductive functions (98). In ruminants, plant extracts can also modify the rumen microbiome, affecting fermentation and potentially reducing methane production. This is important because a balanced rumen environment ensures efficient nutrient absorption, which is necessary for optimal reproductive performance.

### Antioxidative and anti-inflammatory mechanisms

Oxidative stress (OS) significantly affects livestock reproductive health by causing cellular damage in reproductive tissues, impacting fertility and lower reproductive performance. Specifically, they disrupt follicular growth, causing selective atretic follicles with genes associated with inflammation and apoptosis processes highly enriched. In contrast, genes related to the biosynthetic pathway, response to oxidative stress processes, and the glutathione metabolism pathway were downregulated (99). Similarly, Liu et al. (100) showed that FOXO1 expression and granulosa cell apoptosis increase under oxidative stress conditions. In addition, sperm cells contain less enzymatic antioxidants and are susceptible to oxidative stress. Therefore, imbalances in oxidant-antioxidant ratio in sperm cells prompt sperm functional abnormalities and mitochondria dysfunction (12).

Phytogenic extracts are also known for their antioxidative and anti-inflammatory properties. These antioxidant compounds scavenged ROS and protected reproductive and other cells from oxidative damage. They also reduce inflammation by modulating immune responses. A study by Kaschubek et al. (101) explored the antioxidative and anti-inflammatory effects of a phytogenic feed additive (PFA) in porcine intestinal cells (IPEC-J2), comparing it to other phytogenic compounds like grape seed extract, licorice extract, and oregano essential oil, as well as the antibiotic growth promoter tylosin. The findings revealed that the PFA, licorice, and oregano effectively reduced ROS in cells exposed to hydrogen peroxide, demonstrating significant antioxidative activity. Additionally, both the PFA and oregano inhibited the NF-κB pathway, which is crucial for inflammation, leading to a decrease in the expression of inflammatory cytokines such as IL-6, IL-8, and CCL2. These results emphasize the antioxidative and anti-inflammatory benefits of phytogenic, especially PFA and oregano, as potential alternatives to antimicrobial growth promoters in animal feed.

Another research investigated the effects of PFAs, specifically a blend of eucalyptus (*Eucalyptus citriodora*) and poplar (*Populus deltoides*) leaf-meal (EPLM), on the immune response, antioxidant status, and overall health of buffalo calves. The results showed that supplementation with EPLM enhanced the antioxidant status of the calves, increasing the levels of key antioxidants such as reduced glutathione (GSH), catalase (CAT), and superoxide dismutase (SOD) while reducing lipid peroxidation (LPO), which is an indicator of oxidative stress (102). These improvements suggest that EPLM effectively mitigates oxidative damage in livestock. The supplementation also enhanced immune function, evidenced by a higher antibody titer against *Pasteurella multocida* and increased skin thickness following an immune response test. The study concluded that EPLM, rich in phenolic compounds, can enhance buffalo calves’ antioxidative and immune responses, benefiting their overall health and potentially improving reproductive performance by reducing oxidative stress and inflammation. Also, the increase in endogenous antioxidant enzyme activity levels like catalase and superoxide dismutase in the reproductive system supports healthier pregnancies and offspring (102). In addition to the restoration of hormonal imbalance, *Artemisia annua* or sweet wormwood has been reported to significantly reduce the level of oxidative stress and ameliorate spermatogenesis malformations caused by high-fat diet (103).

The role of β-carotene in enhancing ovarian function and ovulation through mechanisms independent of luteinizing hormone-releasing hormone has also been reported. For instance, Arellano-Rodriguez et al. (104) examined the impact of the dietary supplementation of β-carotene on luteal activity and progesterone (P4) production in goats. The β-carotene group showed a trend toward more corpus lutea and significantly higher P4 levels, while luteum volume was similar between the β-carotene and control groups. These findings suggest that β-carotene enhances progesterone synthesis, supporting healthy ovulation and pregnancy maintenance in goats. Similarly, Meza-Herrera et al. (105) also demonstrated that β-carotene supplementation (50 mg/day) increased ovulation rate, total ovarian activity, and lower LH levels compared to controls.

Kaempferol is one of the major groups of flavanols that originated from the aromatic ginger plant rhizomes and can be found in many other plants like fruits and vegetables. It has antioxidant and anti-inflammatory benefits. Santos et al. (106) investigated the impact of kaempferol, alone or with antioxidants (transferrin, selenium, and ascorbic acid), on the *in vitro* culture of sheep secondary follicles and the role of the PI3K pathway. The study showed that the antioxidant-supplemented medium and α-MEM with 1 or 10 μM kaempferol maintained a higher percentage of normal follicles, similar follicular diameter, fully-grown oocytes, and glutathione levels. Specifically, 1 μM kaempferol improved antrum formation and mitochondrial activity, comparable to higher kaempferol levels and the antioxidant mix. Additionally, inhibiting the PI3K pathway reduced these positive effects, indicating kaempferol acts through this pathway. The study concluded that 1 μM kaempferol can replace the traditional antioxidant combination in the culture medium, supporting follicle survival, enhancing mitochondrial function, and promoting oocyte maturation via the PI3K pathway (107).

Quercetin, another bioactive compound in the flavonoid family found in many fruits, vegetables, and teas and shows antioxidative, anti-inflammatory, and antibacterial benefits, has been found to stimulate the FSH hormones that influence ovulation in rabbits and pigs in a dose-dependent manner (108). Conversely, genistein, a phytoestrogen found in soy and other plants, disrupts the estradiol biosynthesis pathway by binding to estrogen receptors, potentially affecting female fertility and health. Studies showed genistein inhibits antral follicle growth, alters steroid hormone production, and causes cell cycle arrest *in vitro*. In mice, preconception exposure to dietary genistein affected fertility, gestation, litter outcomes, and maternal behavior, with prolonged exposure linked to ovarian cysts and altered follicle numbers. Despite the minimal impact on serum hormone levels, these findings highlight potential long-term effects on reproductive health and ovarian function (109). Similarly, daidzein, another phytoestrogen found in soybean has shown to have an adverse impact on the reproductive process in livestock. According to Wu et al. (110), who examined the effect of the phytoestrogen daidzein on blastocyst implantation in rats. Female rats received daidzein during early pregnancy. High-dose treatment of daidzein significantly reduced blastocyst implantation rates and serum GnRH, progesterone, FSH, and LH levels while increasing beta-endorphin levels. These effects were absent in the low-dose group (50 mg/kg). The findings suggest that daidzein’s anti-implantation effects may result from disrupting the hypothalamus-pituitary-gonadal axis critical to implantation.

### Hormonal modulation and fertility enhancement

Phytogenic compounds, derived from medicinal herbs and plants, have been shown to mimic natural hormones and influence reproductive performance in both male and female livestock. The study on *Yucca schidigera* powder (YSP) supplementation in Zaraibi dairy goats showed significant improvements in both reproductive and productive performance. Compared to the control group, goats fed 250 mg and 500 mg of YSP daily exhibited increased body weight, better fertility rates, and enhanced milk production during lactation. YSP intake also resulted in shorter estrus resumption periods, longer estrus durations, improved blood parameters, lower cholesterol, triglycerides, and urea levels, and higher glucose and calcium levels in the treated groups. These findings suggest that YSP supplementation positively influences dairy goats’ reproductive health, milk yield, and overall productivity (111). Similarly, the addition of *propolis* in the diet of pregnant Barki ewes led to improvements in milk yield, composition, and antioxidant enzyme activities. Ewes receiving propolis showed increased milk fat, total solids, and higher plasma immunoglobulin A (IgA) levels, indicating better immune function. The supplementation also reduced oxidative stress, as evidenced by lower malondialdehyde and hydrogen peroxide levels in both ewes and their lambs. Additionally, lambs born to propolis-fed ewes had higher weaning weights, suggesting enhanced growth performance. Overall, propolis supplementation in ewes under arid conditions positively affected milk production, immune status, and lamb growth, highlighting its beneficial effects on livestock health and productivity (80). Another study assessed the effects of *Aegle marmelos* (bael leaf) and *Murraya koenigii* (curry leaf) on estrus induction, ovulation, and conception rates in buffalo heifers with delayed puberty. Heifers given *A. marmelos* and *M. koenigii* exhibited high estrus response, ovulation and conception rates. These suggest that herbal supplementation, particularly the combination of *A. marmelos* and *M. koenigii*, effectively improves fertility in delayed pubertal buffalo heifers (112).

Showing its effects on male fertility, the study on *Symphonia globulifera* root extract in male Wistar rats showed significant fertility-enhancing effects. Oral administration of the aqueous extract, particularly at higher doses, resulted in a dose-dependent improvement in seminal fluid analysis and increased follicle-stimulating hormone (FSH) levels, however, testosterone levels decreased. These findings suggest that *S. globulifera* extract has potential as a natural fertility booster in male rats, enhancing reproductive health without toxicity (113, 114). Research on rabbit bucks’ turmeric, garlic, and moringa extracts demonstrated improved semen quality, reproductive hormone levels, and immune response. Supplementation with these extracts increased key hormones like FSH and testosterone and enhanced hepatic antioxidant activity. Both turmeric and garlic significantly improved semen characteristics, including motility, sperm concentration, and acrosome integrity, while reducing sperm abnormalities. These results highlight the potential of turmeric and garlic as effective dietary supplements to boost semen quality and reproductive health in male rabbits (115, 116).

**Figure 2:**
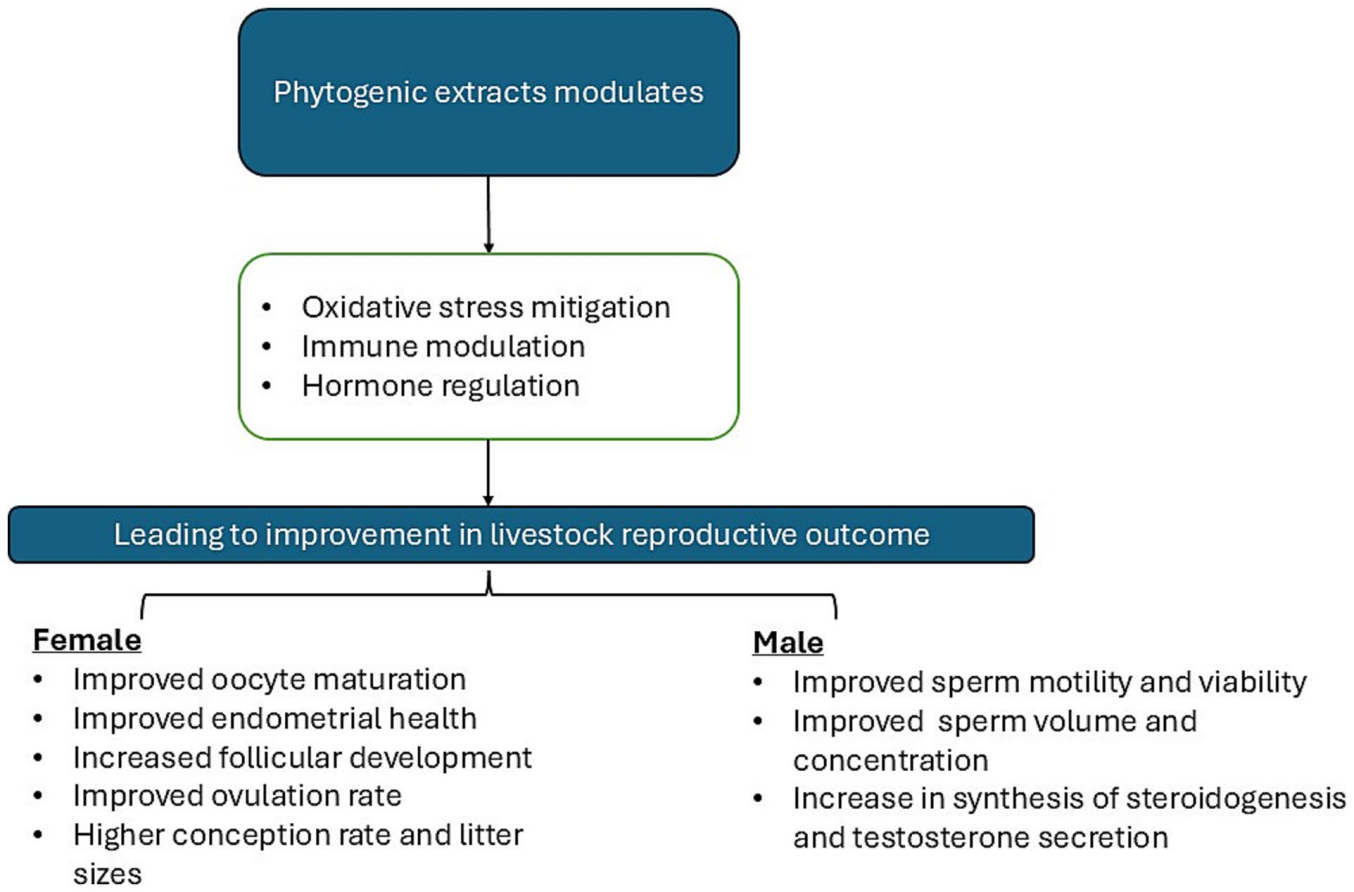
Mechanism of action by which phytogenic extracts modulate reproductive outcomes.

In a study that explored innovative treatments to improve reproductive outcomes using *Momordica charantia* L. (MC) extract, MC extract positively influences hormonal dynamics, particularly by enhancing insulin-like growth factor (IGF-1) levels, which play a pivotal role in the endocrine regulation of fertility. This treatment was associated with improved pregnancy rates in repeat breeder cows, highlighting its potential as a therapeutic option for addressing fertility issues, particularly in animals affected by subclinical endometritis (SCE) (117) (Figure 2).

## Potential risks of phytogenic extracts in livestock reproductive health

Phytogenic extracts are recognized in a positive context for their different pharmacological and beneficial effects, including antimicrobial, antioxidant, anti-inflammatory, and immunomodulatory activities. Importantly, they do not negatively affect growth or feed efficiency, making them valuable as growth-promoting feed additives in livestock production (40, 118). Despite the notable advantages and benefits, it is essential to acknowledge the valid concerns and feedback regarding the appropriate limit of use. Certain phytogenic extracts may contain prohibited substances, such as caffeine, heroin, salicylates, and steroids, which possess drug-like effects that can interact with other dietary components, potentially leading to mild to severe side effects and toxicities (118, 119).

Tannins are a group of high molecular weight polyphenolic compounds known for their astringent properties. They have the potential to enhance reproductive performance, reduce oxidative stress, and improve immune indices in livestock (120). However, the dietary intake of phenolic compounds, including tannins, has been associated with gastrointestinal disturbances. In postpartum buffalo cows fed diets supplemented with quebracho tannin, the total number of ovarian follicles, peripheral progesterone concentration, and conception rate reduced significantly at an inclusion level of 100 and 200 g/(cow·d) (121). A recent scientific review also explored the antinutritional effects of this compound. High levels of tannins negatively impact appetite and are inversely related to the digestibility of organic matter and protein in both ruminants and monogastric livestock due to their binding effects (118). Moreover, a delay in the gastrointestinal passage can result from elevated tannin levels in feed. In monogastric livestock, condensed tannins are recognized as antinutritive substances due to their harmful effect on protein digestibility, which occurs through the binding of dietary and endogenous proteins, including digestive enzymes (122). Research has shown that high concentrations of chestnut tannins lead to liver and kidney toxicity in ruminants. However, no adverse effects were observed on neonatal pigs and rats’ liver, kidneys, stomach, or small intestine in a 28-day trial (120).

While the inclusion of *Antidesma thwaitesianum* Muell. Arg. pomace (containing 98 g/kg saponins) in the diet of lactating cows did not affect nutrient digestibility (123), saponins derived from *Sapindus saponaria*, *Yucca schidigera*, and *Quillaja saponaria* have been shown to decrease *in vitro* digestibility of both neutral detergent fiber and non-structural carbohydrates in dairy cows and rumen fluid (124, 125). Also, the use of tea saponin extract led to a significant reduction in dairy cow milk yield compared to control (126). Similarly, phytoestrogens have been reported to have adverse effects on livestock reproductive performance when consumed in high amounts or after prolonged exposure. For instance, ingestion from grazing on lucerne pasture for a prolonged period has led to anestrus in ewe (127). In heifers, feeding coumestrol and genistein at a concentration of 10 and 100 μg/mL, respectively, have increased the number of immature oocytes (128), however, at 100 μM, the beneficial effect of supplementing genistein in ram diet was lost, resulting in deleterious effect on the functionality and quality of ram sperm (129).

In the context of sperm physiology and preservation, elevated levels of oregano, splinter bean (*Entada abyssinica*), rosemary, and pomegranate have been found to negatively affect certain sperm parameters, potentially compromising sperm functionality (66). *Myrrhinium atropurpureum* and *Cymbopogon citratus* (also known as lemon grass) leaves are native sources of compounds widely used in folk medicine as astringents and antimicrobials or to treat inflammatory conditions and gastric disturbances (130, 131). A recent study indicated that while these phytogenics are rich in monoterpenes, they exhibit cytotoxic effects on swine spermatozoa. The authors observed a complete reduction in sperm motility, membrane functionality, and mitochondrial membrane potential, making them unsuitable as additives for semen extenders (132). Furthermore, the *in vitro* effects and toxicity of *Thymba capitata* (L) cav. and *Rosmarinus officinalis* on the morpho-functional parameters of swine spermatozoa were investigated. *T. capitata* demonstrated a spermicidal effect, reducing total motility at a concentration of 0.2 mg/mL, with complete immobilization of spermatozoa observed at higher concentrations (133).

The effect of phytogenic fed additives has been linked to a reduced villi length and crypt depth in the jejunum and colon of broilers and pigs (134). Manzanilla et al. (135) and Nofrarías et al. (136) reported a decrease in the number of intraepithelial lymphocytes in the jejunum of weaned pigs treated with either antibiotics or PFAs. Anthocyanin pigments and polyphenolics have been shown to reduce the incidence of coronary heart disease, but there remains considerable debate and opinions about their potential role in tumor development (122). Some studies have highlighted the harmful health effects associated with consuming antioxidant supplements. The authors noted that such supplements could potentially lead to increased mortality, particularly with fat-soluble antioxidants like the plant pigment β-carotene and vitamins A and E (tocopherol) (137). Reports suggest that antioxidant supplements had no significant influence on the occurrence of gastrointestinal cancer. However, participants in the antioxidant group developed gastrointestinal cancers during the follow-up period (138). The findings challenged the prevailing belief that antioxidants are beneficial in preventing gastrointestinal cancer, cardiovascular diseases, and various other health conditions (137, 138). It is essential to recognize that although these observations relate to human participants, the potential side effects of these phytogenic products in livestock reproduction may arise at certain doses.

Instances of residue concerns related to phytogenic extracts have been noted. Phytogenic extracts often contain bioactive compounds such as alkaloids, flavonoids, and terpenoids, which may accumulate in animal tissues and products. For instance, glucuronic and sulfate metabolites of carvacrol and thymol were detected in the blood and kidney of swine fed with PFAs (133). PFAs can interact with other feed additives, potentially leading to the accumulation of phytogenic residues. Although many phytogenic compounds degrade during metabolism, their stability can vary significantly, and residues may persist depending on the specific compound and the animal’s metabolism. It appears that the residues may be non-toxic; however, the safety of these residues for long-term human consumption is still not well researched. It is essential to prioritize the safety of phytogenic products and the conditions under which they demonstrate biological effects when evaluating their use as feed additives in livestock. Although phytogenic extracts are generally considered safe, limited regulatory frameworks and the lack of established maximum residue levels may hinder their safe application in animal husbandry. Additional research is necessary to develop safety guidelines, residue detection methods, and regulatory standards (Table 2).

**Table 2:** Representative potential risks of high dietary inclusion or exposure to phytogenic extracts.

Plant metabolites	Negative impact on livestock	Reference
Tannins	Gastrointestinal disturbances Reduction in progesterone concentration and conception rate Digestibility of organic matter and protein	(120–122)
Phytoestrogens	Anestrus Decrease in the functionality and quality of ram sperm Fetal loss Alteration in reproductive hormones, spermatogenesis, sperm capacitation, and fertility Decrease in the functionality and quality of ram sperm	(127–129)
Saponins	Decrease in vitro digestibility Reduction in milk yield	(126)

## Conclusions and future directions

Phytogenic extracts have shown significant promise in improving reproductive health across various livestock species by enhancing gut health, reducing oxidative stress, and modulating hormonal functions. Through mechanisms such as improving nutrient absorption and modulation of gut microbiota, phytogenic extracts help optimize animal health, directly influencing reproductive outcomes. Notably, their antioxidative and anti-inflammatory properties have been shown to mitigate oxidative damage, further supporting fertility and reproductive success. The hormonal modulation observed with various PFAs suggests that these compounds can enhance both female and male fertility, contributing to overall productivity. As livestock production systems shift toward reducing antibiotic usage, phytogenics offer a promising alternative, functioning through mechanisms that align with the biological needs of animals, supporting a holistic approach to sustainable agriculture. Despite these promising results, several challenges remain. The mechanisms by which phytogenic compounds influence reproductive health need further elucidation, particularly in terms of their dose-dependent effects, the long-term safety of their residues, and their interaction with the gut microbiome. Research into the synergistic effects of combining different phytogenics could also provide more insights into optimizing their efficacy. In addition, *in vivo* studies that focus on the impact of phytogenic extract supplementation on fertility rate, conception rate, and litter size, which directly impact livestock economics, should be conducted.

Furthermore, regulatory frameworks must evolve to accommodate the increasing use of these plant-derived compounds, ensuring that their benefits are maximized while minimizing potential risks. Moving forward, further research should focus on optimizing delivery methods, such as microencapsulation, to enhance the bioavailability of active compounds in target organs. Additionally, exploring the interaction between phytogenics and other therapeutic treatments will be key to understanding their full potential as sustainable, non-antibiotic alternatives for improving livestock health and productivity. Collaborative efforts between scientists, regulatory agencies, and commercial entities will be crucial in harnessing the full potential of phytogenics for future agricultural and reproductive health advancements.
