# Correction: Mechanisms by which phytogenic extracts enhance livestock reproductive health: current insights and future directions

**DOI:** 10.3389/fvets.2025.1720253

**Published:** 2025-11-21

**Authors:** Adedeji O. Adetunji, Jacqueline Price, Henrietta Owusu, Esiosa F. Adewale, Precious Adedayo Adesina, Tolulope Peter Saliu, Zhendong Zhu, Christian Xedzro, Emmanuel Asiamah, Shahidul Islam

**Affiliations:** 1 Department of Agriculture, University of Arkansas at Pine Bluff, Pine Bluff, AR, United States; 2 Department of Biology, University of Louisville, Louisville, KY, United States; 3 Graduate School of Integrated Sciences for Life, Hiroshima University, Higashi-Hiroshima, Japan; 4 Department of Physiology, College of Medicine, University of Kentucky, Lexington, KY, United States; 5 College of Animal Science and Technology, Qingdao Agricultural University, Qingdao, China; 6 Laboratory of Food Microbiology and Hygiene, Hiroshima University, Higashihiroshima, Japan

**Keywords:** plant extract, hormone regulation, immune modulation, oxidative stress mitigation, reproductive health, phytogenic extracts, bioactive compounds, livestock reproduction

Author Precious Adedayo Adesina was erroneously assigned to affiliation “National Center for Advancing Translational Sciences, Division for Pre-Clinical Innovation, National Institutes of Health, Bethesda, MD, United States”. This affiliation has now been removed for author Precious Adedayo Adesina.

Affiliation “Graduate School of Integrated Sciences for Life, Hiroshima University, Higashi-Hiroshima, Japan” was omitted for author Precious Adedayo Adesina. This affiliation has now been added for author Precious Adedayo Adesina.

The **Conflict of interest** statement was erroneously given as “The authors declare that the research was conducted in the absence of any commercial or financial relationships that could be construed as a potential conflict of interest.”

The conflict of interest statement has been corrected to:

PA was employed by National Institutes of Health.

The remaining authors declare that the research was conducted in the absence of any commercial or financial relationships that could be construed as a potential conflict of interest.

The original version of this article has been updated.

